# Do deepfake videos undermine our epistemic trust? A thematic analysis of tweets that discuss deepfakes in the Russian invasion of Ukraine

**DOI:** 10.1371/journal.pone.0291668

**Published:** 2023-10-25

**Authors:** John Twomey, Didier Ching, Matthew Peter Aylett, Michael Quayle, Conor Linehan, Gillian Murphy

**Affiliations:** 1 School of Applied Psychology, University College Cork, Cork, Ireland; 2 LERO, Science Foundation of Ireland, Limerick, Ireland; 3 University of Heriot Watt, Edinburgh, United Kingdom; 4 CereProc Ltd, Edinburgh, United Kingdom; 5 Centre for Social Issues Research and Department of Psychology, University of Limerick, Limerick, Ireland; 6 Department of Psychology, School of Applied Human Sciences, University of KwaZulu-Natal, Pietermaritzburg, South Africa; King Abdulaziz University, SAUDI ARABIA

## Abstract

Deepfakes are a form of multi-modal media generated using deep-learning technology. Many academics have expressed fears that deepfakes present a severe threat to the veracity of news and political communication, and an epistemic crisis for video evidence. These commentaries have often been hypothetical, with few real-world cases of deepfake’s political and epistemological harm. The Russo-Ukrainian war presents the first real-life example of deepfakes being used in warfare, with a number of incidents involving deepfakes of Russian and Ukrainian government officials being used for misinformation and entertainment. This study uses a thematic analysis on tweets relating to deepfakes and the Russo-Ukrainian war to explore how people react to deepfake content online, and to uncover evidence of previously theorised harms of deepfakes on trust. We extracted 4869 relevant tweets using the Twitter API over the first seven months of 2022. We found that much of the misinformation in our dataset came from labelling real media as deepfakes. Novel findings about deepfake scepticism emerged, including a connection between deepfakes and conspiratorial beliefs that world leaders were dead and/or replaced by deepfakes. This research has numerous implications for future research, societal media platforms, news media and governments. The lack of deepfake literacy in our dataset led to significant misunderstandings of what constitutes a deepfake, showing the need to encourage literacy in these new forms of media. However, our evidence demonstrates that efforts to raise awareness around deepfakes may undermine trust in legitimate videos. Consequentially, news media and governmental agencies need to weigh the benefits of educational deepfakes and pre-bunking against the risks of undermining truth. Similarly, news companies and media should be careful in how they label suspected deepfakes in case they cause suspicion for real media.

## Introduction

Synthetic media are a type of audio-visual media which has been partly or fully generated/modified by technology [1]. Deepfakes are a new form of synthetic media which interpolates artificially generated media into an existing video, often with intent to imitate or mimic an individual. Researchers and commentators have argued that deepfakes have the potential to undermine truth, to spread misinformation and to undermine our trust in information, media and democracy [2]. The increasing prevalence of fake videos could undermine what we know to be true [3]. Specifically, academic researchers believe that deepfakes could create a situation where fake videos are believed to be real and conversely, where real videos are denounced as fake. Fears of deepfakes being used to spread disinformation have been realised during the Russo-Ukrainian war. We have seen fake videos of both Russian President Vladimir Putin and Ukrainian President Volodymyr Zelensky, as well as many satirical and entertainment uses of deepfakes around the crisis. Our paper seeks to provide empirical evidence for the hypothesised and forecasted harms of deepfakes to truth and knowledge. The aim of this paper is to understand the nature of deepfake discourse on social media in the context of the Russo-Ukrainian war.

In this paper, we analyse Twitter discourses around deepfakes in relation to the Russo-Ukrainian war by carrying out a thematic analysis on relevant tweets during the first months of the 2022 invasion. Our study is the first empirical analysis carried out on the use of deepfakes in wartime misinformation and propaganda. As deepfake technology becomes increasingly accessible, it is important to understand how such threats emerge over social media. Understanding the current threats of deepfakes will have implications in how social media companies and academic researchers deal with harmful deepfakes online. Understanding how the threats of deepfakes emerge online is a significant step in learning how to mitigate their harms. The current paper also has numerous implications for academic research on deepfakes. Our research explores the epistemic harms of deepfakes in practice, as opposed to the theoretical discussions of the concept in academia [4]. We also provide a non-exhaustive timeline of the use of deepfakes and other synthetic media in the Russo-Ukrainian war. The provided timeline is important as both a record of the uses and the impact of deepfakes during the Russian invasion of Ukraine and as a means to gauge the type and quality of synthetic media content created during the conflict.

### Background: Deepfakes and misinformation

Deepfakes are an emerging form of synthetic media with potentially harmful implications for truth and democracy [5]. While there is not a consistent definition of the technology, deepfakes are generally understood as audio-visual media which have been manipulated using artificial intelligence technologies [6]. Deepfakes most often consist of videos where a fake “face” constructed by artificial intelligence has been merged with an authentic video. The result of this is a realistic, mimetic recording of someone doing and saying things which they never did or said [7]. Deepfakes were brought to media attention in early 2018 when a reddit community (r/deepfakes), used artificial intelligence to swap celebrities faces into pornographic videos [8]. The potential harms of deepfakes include the erosion of trust in institutions, democracy and journalism [9]. Some journalists have even hypothesised that deepfake misinformation could lead to a world leader declaring nuclear war over deepfake videos [10].

Concerns about deepfakes have centred primarily on their capacity to deceive. Misinformation and disinformation refer to false information utilised to mislead people. The difference between the two concepts is that disinformation is done purposely, while misinformation is not [11]. Deepfakes have the capacity to be used for both misinformation and disinformation by depicting events that never happened [12]. While deepfakes are usually created with deceitful aims, they are often shared online by people who believe them to be unedited videos. For example, in a study carried out by Ahmed [13] in the USA and Singapore, it was found that over 30% of their sample had inadvertently shared a deepfake. Quantitative research around deepfakes has broadly focused on individuals’ ability to detect deepfakes [14], and factors which lead to accidental sharing of deepfakes [15]. In contrast, qualitative research has focused on the supposedly positive elements of deepfakes on social media, focusing on entertainment videos on YouTube [16, 17]. There has also been some quantitative research into the effects of deepfakes on trust. Vaccari and Chadwick [18] found that deceptive deepfake videos were significantly more likely to elicit uncertainty compared with the same video containing an educational disclaimer. However, they did not compare this to a control group who hadn’t been exposed to a deepfake, which poses a broader theoretical question as to whether or not educational deepfakes may be harmful to truth in themselves and if the very existence of deepfake technology may pose a threat to truth.

The broader theoretical concern as to how the very existence of deepfakes may cultivate misinformation is exemplified by the liar’s dividend. As described by Chesney and Citron [2], the liar’s dividend refers to a situation in which people may avoid accountability for real audio and video evidence as a result of public scepticism. As the public becomes more and more aware of novel video manipulation technologies, real video evidence will be increasingly denounced as fake. For example, in a hypothetical situation, where a politician is faced with compromising video evidence, they could deny a factual incriminating video of themselves as a deepfake. The prevalence of the liar’s dividend also increases proportionately with the public’s awareness of deepfakes [2]. Encouraging scepticism towards visual media may unintentionally have the adverse effect of making it more likely that people will flatly deny real video evidence with which that they are presented [19]. While pre-emptive misinformation interventions, known as pre-bunking, can be used to combat other forms of misinformation, pre-bunking deepfakes may only increase their epistemic harms [20]. Quantitative research into the efficacy of deepfake misinformation interventions has found that while interventions improved people’s ability to identify deepfakes it also reduced participants’ ability to correctly identify real media by 9% [21]. While the capacity of deepfakes to undermine people’s ability to distinguish truth has thus been shown in experimental contexts, there is a lack of research qualitatively exploring how this epistemic distrust may manifest online.

The liar’s dividend demonstrates deepfakes’ potential to encourage deniability of real video evidence and harm the work of journalists, governments, and the legal system [22]. The doubt that deepfakes cast on the truthfulness of video evidence has been described as the biggest epistemic threat of the 21^st^ century [23]. Interestingly, despite these drastic projections made by commentators when deepfakes first emerged, there have not been many high-profile or large-scale uses of deepfakes for misinformation purposes. This has led some academics to consider the epistemic harms of deepfake overblown and to suggest that deepfakes may conversely have a positive effect on trust due to an increased scepticism towards dubious online sources and a move to more verifiable content [24]. In practice, most existing uses of the technology revolve around harassment and bullying, particularly image-based abuse of women [8]. Any uses of deepfakes in the political sphere have largely been benign, such as a Belgian political message spoken by a deepfake of Donald Trump, which was used to highlight climate change [25]. The use of deepfakes on social media during the Russian invasion of Ukraine indicates that this may be changing.

### Deepfakes, disputed deepfakes and other synthetic media during the Russian invasion of Ukraine

The Russo-Ukrainian war began in 2014 with the Russian invasion and annexation of Crimea [26]. In the years after this, Ukraine saw increased acts of aggression initially by Russian-proxy forces and Russian backed separatist militias in the Donbas region of Ukraine [27]. Later responses to Ukraine’s attempts at a counterattack were foiled by covert troops and weapons from the Russian armed forces [28]. In the later months of 2021, Russian forces were amassed at the two countries’ borders under the pretence of a training exercise. At this time, Russian governmental figures made incorrect and disingenuous claims of widespread Russophobia and Nazism in Ukraine [29]. This was further fuelled by a controversial address made by the Russian president Vladimir Putin which incorporated similar “conspiracy theories and lies” to attempt to undermine the history and existence of Ukraine as a Sovereign state in its own right [30]. On the 24^th^ of February 2022, the armed forces of Russia launched a full-scale military invasion of the country of Ukraine [31]. These actions, along with the brutality of the Russian invaders, drew widespread condemnation from many governments and organisations both within Europe and worldwide [32].

**Fig 1:**
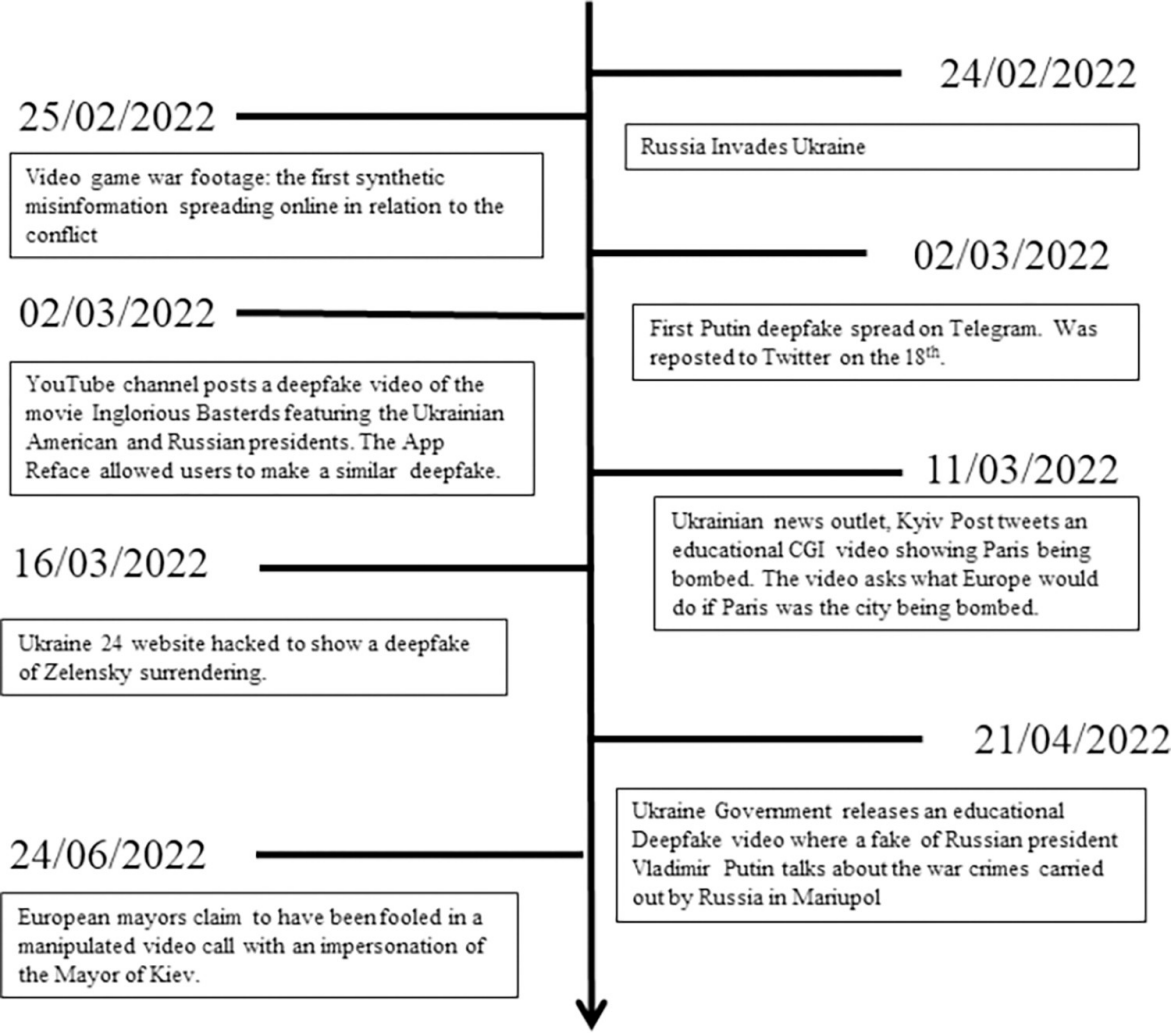
Timeline of the use of deepfakes/synthetic media in the war. Images via Twitter, YouTube, Facebook, Reddit and Deutsche Welle.

Public outrage and interactions with the Russo-Ukrainian war have been mediated by social media platforms and technology. Cell-phone journalistic practices mean that first-hand accounts of the atrocities of the conflict are readily available online [33]. Internet activism has meant that individual users of the internet have been able to co-ordinate DDOS attacks on Russian websites [34]. While modern technologies have facilitated the spreading of first-hand accounts of the brutality of the war, they have also been used to spread misinformation and propaganda [35]. The Russo-Ukrainian war has seen for the first time, the use of deepfake technology in wartime propaganda and misinformation. Fig 1 provides an overview of deepfakes (as well as similar synthetic media that have been misidentified as deepfakes) used in the Russo-Ukrainian war during the first four months of the invasion. In this section we will discuss the key events relating to deepfakes, contested deepfakes and other synthetic media in the war in more detail.

#### Synthetic video footage of a Ukrainian fighter jet

Misinformation in the early days of the war was not characterised by deepfakes. Instead, most misinformation consisted of the misuse of videos from the earlier conflicts in Ukraine. Notably, the second day of the war saw the spread of video game footage as evidence of the urban myth fighter pilot “The Ghost of Kyiv”. This video footage was taken from the video game Digital Combat Simulator. It was spread around social media during the first few days of the invasion purporting to show a Ukrainian fighter jet [36]. The Ukraine Department of Defence even tweeted the viral footage. This serves as the first known example of synthetic media concerning the Russian invasion of Ukraine.

#### Deepfake of Putin announcing peace with Ukraine

In early March a deepfake of Russian president Vladimir Putin emerged, showing the Russian president announcing peace with Ukraine. The deepfake was first published online in the first week of March on the reddit r/sfwdeepfakes and r/ukraine communities [37]. It was posted with an acknowledgment that it was fake and the user who submitted this video claimed to have found it on the social media site Telegram and added their own subtitles. The deepfake was then published on Twitter on the 18^th^ of March. This is the version that was reported on by news agencies. The version posted on Twitter did not contain subtitles, suggesting it may have come from Telegram. This deepfake was unique out of the major examples of the technology in the conflict as it has been suggested that the audio was also generated using AI [38].

#### Deepfake of Zelensky surrendering to Russia

In the months leading up to the invasion, the Ukrainian government warned of the potential for Russia to employ a deepfake of Zelensky surrendering to the Russian Government [39]. On the 14^th^ of March 2022, a Ukrainian news website was hacked to display a deepfaked message of Zelensky surrendering. Concurrently, the News Ticker of Ukraine24’s television channel was changed to read a similar message. Despite media coverage of this event accusing the government of Russia of orchestrating the hack, they have not claimed responsibility to date, nor has any significant evidence been found indicating their involvement. This event is significant as it is one of the first cases of a deepfaked politician being intentionally used to spread disinformation. The quality of the deepfake was poor and quickly debunked by Zelensky himself. However, this incident highlights the potential harms of deepfakes. The usual indicator of truthfulness and trustworthiness of online information, the source of the video, was undermined by the hack. If the video had been more realistic and more widely believed, it may have had a more harmful impact.

#### Educational deepfakes from the Ukrainian government and news media outlets

On the 21^st^ of April, the government of Ukraine released an educational deepfake video in partnership with Reface, a Ukrainian AI company. Titled “Putin telling truth, huh? Well, we tried to imagine what he’d say if he did”, the footage showed a deepfake Putin walking around the Ukrainian city of Mariupol and describing the war crimes carried out by the Russian forces [40]. A similar non-deepfaked video was posted the month before consisting of a CGI video of Paris being bombed, asking how European citizens would feel if their cities were being attacked. These two examples are relevant as they show the educational and political communication uses of synthetic media and deepfake technology.

The Reface app also allowed users to swap their faces into Zelensky’s speeches, or onto images of Ukraine soldiers [41]. The app encouraged users to faceswap themselves and Putin into a scene from the Quentin Tarantino movie “Inglorious Basterds” in which a Nazi is violently beaten to death by an American soldier. Versions of these videos including Zelensky as the American soldier also went viral online [42]. This is one example of the numerous online videos recasting the two presidents into Hollywood movies and other satirical or humorous videos. Most of these videos involved using AI to insert the Russian and Ukrainian presidents into comedic movie scenes or dancing/lip-synching to music. Oftentimes, political figures in the war would be recast as famous movie villains or heroes. These videos were clearly satirical. While intended to influence, they were obvious parodies and probably not intended to deceive.

#### European mayors receive possible deepfake video call from the mayor of Kyiv

On the 24^th^ of June, three European mayors came forward as victims of a deepfake video call. The mayors of Berlin, Madrid and Vienna had believed they were talking to the mayor of Kyiv, Vitali Klitschko [43]. After a certain point, odd and seemingly nonsensical questions alerted the politicians that this was not the real Vitali Klitschko. This potential deepfake is both unverified and contested, with some suggesting that the hack does not contain deepfaked footage and the video consisted of reused video clips [44]. Two Russian comedians, Lexus and Vovan have claimed responsibility for the video, but haven’t stated how the video was manipulated [45]. This case is quite similar to an incident in 2020, in which many mayors decried the use of a supposedly deepfaked video call with Leonid Volkov. It was in fact a prank call also orchestrated by Lexus and Vovan. The supposed deepfake was no more sophisticated than make-up and cleverly chosen, obfuscating camera angles [46]. The pair of comedians, who are notoriously pro-Putin, have attracted attention with similar actions during the Russo-Ukrainian war, imitating Zelensky during an audience with noted author J.K.Rowling [47].

While it is possible to deepfake a face in a video-call with software such as DeepfaceLive [48], the software to accomplish this is in its infancy and while capabilities have advanced significantly in the last two years, it still often requires significantly more work to get to a lower level of quality than traditional deepfakes. It is more likely that the video call used either manually (non-deepfaked) stitched together clips from a previous interview or lip-syncing technology (which is considered by some as a type of deepfake). Evidence for this is the fake mayor’s insistence on using Russian despite their fluency in German and how the video frames seem to come from an earlier interview from the mayor. Nonetheless this is an example of the capabilities of fake videos in warfare and the current vigilance towards potential deepfakes. It also highlights how news media and governmental agencies can label traditionally manipulated videos as deepfakes, contributing to a fear of the capabilities and prevalence of the technology.

### The current study

The Russo-Ukrainian war has highlighted the impact of novel misinformation technologies on online media spaces. We have seen the realisation of many fears around deepfake technologies, in which deepfaked presidents have been used to affect public opinion regarding an active war. However, it is unclear how this new form of wartime misinformation was perceived online at the time, and what harms were caused. It is integral to assess this, so governments, social media organisations, and other stakeholders are able to understand the harms deepfakes may cause to social media discourse in present and future conflicts. We used qualitative analysis to explore Twitter users’ comments on the topic of deepfakes during the Russo-Ukrainian war. This data provides a unique opportunity to assess the potential harms of deepfakes in a real-world situation, as well as assessing evidence for some of the fears around deepfakes, such as the liar’s dividend and the harms to epistemic truth.

Our research questions are as follows:

1. How have people responded on Twitter to deepfake content during the Russian invasion of Ukraine?
2. Does Twitter discourse around deepfakes provide real-world qualitative evidence of the epistemic harms of deepfake technology which have been previously theorised and experimentally studied?

## Methods

### Data collection

The decision to study Twitter was because the majority of the most prominent cases of deepfakes (and similar synthetic media) identified in our timeline involved the social media platform. We used the Twitter developer API to capture tweets relating to deepfake content from the 1st of January 2022 to the 1st of August. The API was accessed using a python program based on one from the Twitter GitHub page [49]. The search term used was “(deepfake Zelensky OR deepfake Ukraine OR deepfake Putin)–is:retweet”. We excluded retweets in our search as they would have been duplicates of an original tweet. We extracted the textual content, the date, and the tweet ID of each tweet into a CSV file. Our final dataset included tweets in English, German, and French. This was done to consider a broader range of western European perspectives rather than a purely English-language dataset, particularly considering the salient German language discourse around the Klitschko video call. Furthermore, these were languages that two members of the research team had experience with, which avoided the pitfalls of relying on purely machine-based translation. Russian and Ukrainian language discourse were excluded out of respect to those involved in the war, to reduce the chance of researcher harm resulting from exposure to sensitive personal and combat footage being included in the dataset, and because we are primarily concerned with the discourse around the war and not the experiences of the war itself. This decision was also informed by challenges with searching the Twitter API for multiple search terms in different languages, the increased likelihood of mistranslation due to the team’s lack of fluency in either Ukrainian or Russian, and issues with sample bias due to the comparative unpopularity of Twitter in Eastern Europe. Similarly, we avoid making claims as to whether tweets were posted by Ukrainian or Russian Twitter users, considering the existence of Russian troll farms which assume false identities and automatically generated accounts to spread misinformation [50]. Any claims made in online research regarding the participants or victims of this war faces issues around the prevalence of these appropriated identities. Research on the actual experiences of Ukrainian nationals with deepfake disinformation during the invasion requires careful ethical consideration and knowledge of identity which cannot be securely gained by this type of broad qualitative research of an online dataset.

As with all qualitative research, is also important to consider the biases and positionality of the research team. It is impossible to make claims to the demographics of the dataset, except that both it and the research team broadly consisted of privileged voices from western Europe and north America. Our research questions do not focus on the specific experiences of people involved in the war with deepfakes, as the specific demographics and anonymity of Twitter reduce any claims to the national identities of any of the participants. While we have justified our decisions to work on this demographic, it does highlight possible alternative avenues for the analysis of deepfake content on social media. Any qualitative research on the experiences of the Ukrainian people with deepfake disinformation (such as the Zelensky deepfakes) would require a different mode of analysis, and a different method of data collection which would avoid the risk of studying false identities online.

In total, 4869 tweets were extracted between the 1st of January 2022 and the date of data extraction, the 28th of August 2022. The extracted dataset was saturated with articles from news media outlets. 1984 tweets were excluded as they consisted of links to news articles with news headlines and quotes and no other relevant reactions and commentary. Similarly, a further 212 only consisted of links to images and videos with no significant textual content. 590 of the posts were excluded for being duplicates, usually consisting of spammed posts. While we aimed to reduce the number of fake posts in our datasets, we limited ourselves to excluding tweets which were obviously spam as further measures of bot-detection would run risks of excluding real data. 691 tweets were excluded because they were in languages not included in our inclusion criteria such as Spanish. Overall, 1392 tweets were included for analysis. 84% of these were English, 12% were German and 4% were French. This is generally unsurprising considering the use of English language search terms. The high prevalence of German tweets in the dataset is most likely a result of the fake Vitali Klitschko video call which affected two German-speaking cities. During the analysis it was found that 161 of these didn’t contain enough lexical density to be used in our analysis because they for example only consisted of self-promotion or spreading news without commentary. In total, our data collection provided 1231 tweets to be analysed. Due to ethical considerations, any links to Twitter pages or attachments were replaced with summaries after the analysis.

### Prevalence of deepfake discourse during the start of the war

**Fig 2:**
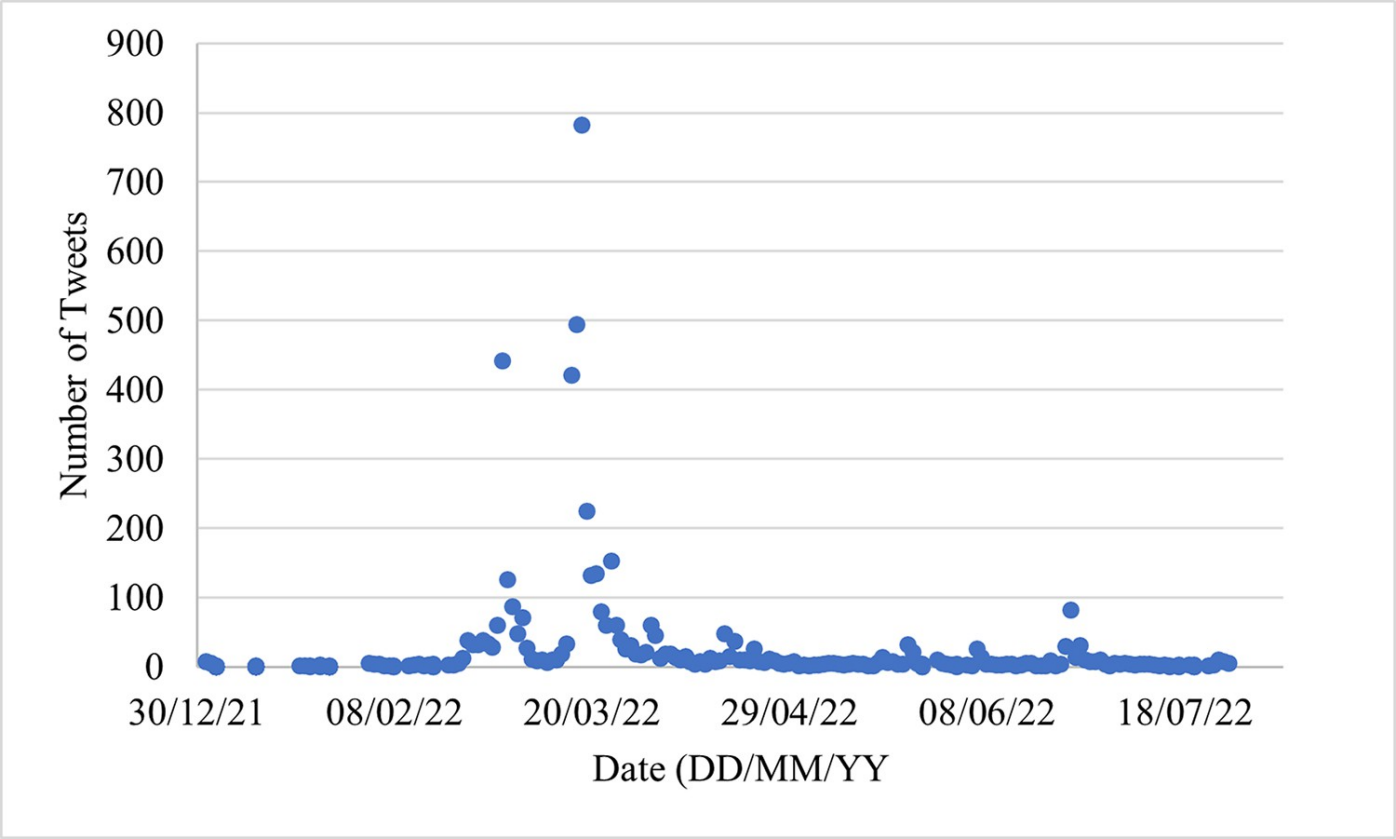
Prevalence of tweets over time in the dataset.

Deepfake related content steadily grew during the week leading up to the war and the first few weeks of the war (Fig 2). This wave of deepfake discourse peaked on the second of February, with 441 tweets being extracted on this day. This largely is the result of spam content and news coverage of the US and Ukraine’s warnings of deepfake content. The largest number of tweets occurred between the 16th to the 18th of March, correlating broadly with the release of the fake Zelensky surrender video and news coverage of the supposed Putin deepfake. A smaller peak occurred during the 4th to 26th of April coinciding with the supposed deepfake video call between Klitschko and the European mayors.

### Ethics

Ethical issues with researching Twitter have been well-established in prior literature. Our ethical processes were designed to mitigate the harms associated with online research, particularly the inability to gain informed consent. Specifically, we followed relevant academic ethical guidelines [51–53] as well as the relevant Twitter developer and privacy policies. We used anonymisation and non-traceability as a means of mitigating this risk. Despite existing as a public online platform that is open to inquiry by researchers, Twitter users still deserve care and anonymity when being studied online, especially in ethnographical research where informed consent cannot be easily gathered. Similarly, many Twitter users are not aware that their content is not only open to use by researchers and businesses, but actively encouraged to be used in this context, and often used in a way which is traceable back to their account [53]. Because of this, it is important to make sure users are not publicly identifiable from research papers.

To ensure users’ anonymity and non-traceability, any examples of tweets in this paper have been replaced with fully fictionalised tweets which represent broadly the original data. Ethical approval to carry out this research was granted by the Social Research Ethics Committee of University College Cork.

### Data analysis

We used and inductive, reflexive thematic analysis [54] to understand and illustrate patterns in our data. We judged thematic analysis as the most appropriate way to address the data set, since the approach is flexible, allowing for many different levels of interpretation. Moreover, there is no existing well-defined theory of how people understand and respond to deepfakes that could guide a more deductive or theory-driven approach. The thematic analysis broadly followed the six steps outlined by Braun and Clarke [55]. Initially, while applying the inclusion and exclusion criteria, we familiarised ourselves with the data, drafting up a series of rough codes in a text document. We then used NVIVO software to descriptively open code the data. Once open coding was complete, we carried out a number of iterative rounds where we sought to meaningfully define observed patterns in the codes. Specifically, we first arranged the codes into small groupings and sub-themes within NVIVO, and then worked on developing larger overarching themes. The majority of this work was done by the primary researcher (White-Irish, Male), with regular consultation with the broader research team throughout the process to iterate on codes and themes. We chose to have one primary researcher do this work as the research often required making judgements to the truth or falsity of deepfake claims and the primary researcher was most aware of both the news media within the dataset (for exclusion of news headlines) and the specific cases of deepfakes used in the war. This was an inductive and iterative process. For example, while initially we approached formulating the codes as a typology of responses to deepfake misinformation, but we found that the range of novel themes and usages of deepfake content outside of misinformation necessitated a broader and more comprehensive set of themes.

After generating and refining a set of three themes, we carried out further analysis, borrowing methodologies from discourse analysis [56], which allowed us to critically appraise the social constructions behind the textual data [57]. We considered how the tweets reflected and drew meaning from the real-world incidences of deepfakes and we incorporated discursive methodologies such as the use of framing and interpretative repertoires. Analysing power relations, positioning, and comparing the discourse in individuals’ tweets such as in critical thematic analysis [58] was impossible due to the anonymity of our dataset, the character limit of tweets and the lack of conversational context. While this research did not involve a content analysis [59], we include the number of references to give the reader an idea of the prevalence of themes in our interpretation of the dataset.

During the writing up process, to ensure the validity of the final quotations, the analysis was written up with the actual quotes before those quotes were then replaced with paraphrased versions. We chose to anonymise the quotes during at the end of the write-up process to ensure that the fictionalisation not affect the analysis. The full research team refined these quotes to ensure they adequately represented the original quotations which included utilising similar typos, using synonyms and rewording the order of sentences. This was done to accurately reflect the original tweets and how they draw from the “new language” of online discourse [60]. The methodology was reported in this paper according to the Consolidated criteria for reporting qualitative research (COREQ) checklist [61], though this had to be adapted due to the unique participant and methodologies of online qualitative studies in contrast to research carried out using in-person interviews.

## Results

Our analysis produced three main themes of online content related to deepfakes and the Ukraine crisis; 1) deepfakes and misinformation, 2) deepfake fuelled scepticism, and 3) non-misinformation related deepfake discourses. The first and third of these are relevant to our first research question: understanding how people reacted to deepfakes online. The second of these, addresses our second question showing epistemic distrust as a result of deepfakes. Each of these will be discussed in the following section using fictionalised versions of the quotations from the dataset.

### Deepfakes and misinformation (interactions with news articles, deepfakes in warfare, solicitation of deepfakes)

A significant portion of our dataset consisted of commentary and general reactions to deepfake-related news articles (n = 246). There was a disproportionate amount tweets which consisted entirely of news articles in the dataset (which were excluded) in comparison to the deepfake discourse that was included for analysis. Users often emotionally reacted to the news about deepfakes with negativity (n = 29), worry (n = 27), shock (n = 6) and confusion (n = 6), mostly targeted towards news about the deepfake Zelensky surrender. This highlights how reactions to news articles seemed to overshadow independent discourse relating to deepfakes. Many tweets in the dataset reacted to the news of the deepfake as something that had been expected (n = 36), highlighting the many attempts of pre-bunking carried out by the Ukraine government.” *Well this confirms the warning that Russia would use a deepfake of Zelensky surrendering*”. The reaction to deepfakes in the war was generally negative. “*Deepfake tech has arrived*, *and it’s absolute madness*” Many tweets sought to explain deepfakes and deepfake news for other users, spreading information about their prevalence and defining the technology. Some spoke to the anecdotal experiences they had had with deepfakes during the war (n = 3). A significant portion revolved around the technical appraisal of the technology, often reflecting on their poor quality (n = 109). Specifically, many users criticised the quality of the Zelensky deepfake, which users felt didn’t live up to warnings of deepfakes or their own expectations of the technology. *“You’ve seen the deepfake of Zelensky*, *now we’ve seen a deepfake Putin declare surrender*. *This is clearly better quality than the Zelensky cheapfake but it’s still poorly made*. *Unfortunately*, *the technology will get better with every advance of the technology”*. Some Twitter users reacted positively to deepfakes used during the war, particularly those used for entertainment value (n = 5), especially demeaning videos such as those putting Vladimir Putin’s face onto Gollum from the Lord of the Rings movies. Other people positively reacted to the deepfakes used for misinformation (n = 15), especially towards those made of Vladamir Putin. In some cases, they framed them as revenge for the Zelensky deepfake.*”In an outstanding retaliatory move by pro-Ukraine VFX artists*, *a deepfake of the Russian president has been released as a response to the deepfake of the Ukrainian president*. *Gives insight into the future and how edited videos can be used as a response to deepfakes*.*”*

One element of deepfakes that tweets seemed to engage in strongly was the potential of deepfakes in warfare. Deepfakes were seen as a new form of weapon (n = 28) or a new kind of propaganda (n = 10). *“You’d think deepfakes are harmless*, *if you’ve only seen silly videos of deepfaked Keanu Reeves*. *Unfortunately deepfakes can be a new and vicious type of propaganda*. *We’ve seen it now with deepfakes of the Russian and Ukrainian leaders”* In January and early February, people feared deepfakes would be used as a false flag to start the war (n = 22). “*Russia have plans to use a deepfake video to justify an invasion of Ukraine*. *Before making a statement I need some time to process this*”. As a response to this, many tweets also focused on detecting and preparing for deepfakes (n = 17) and criticising government bodies for perceived inaction (n = 20). In particular, German language tweets tended to express doubts about their own politicians’ abilities to detect deepfakes. The Klitschko hoax phone call was perceived as a worrying or embarrassing indicator of government cyber-security. *“Some political actors are very behind the times*. *The stupidity of using technology which is not compliant with modern internet safety protocols*. *There’s no excuse when there are means of authenticating video calls”*. The doubts over cyber-security led to many users encouraging stricter media controls (n = 14). These often framed the rise of deepfakes and misinformation as a justification of the decision made by the Ukrainian government to take over private news agencies. “*Ukraine is completely justified in taking over news companies*, *it is very important to stop Russia’s misinfo*. *Putin used a deepfake of Zelensky surrendering and has used their propaganda news to rig a US election*.” Some also demanded significant improvements in governments cyber-security or the banning of deepfake technologies altogether.

A disturbing trend in the dataset involved Twitter users, often jokingly, requesting that people create deepfakes of world leaders in incriminating or humorous defamatory videos. These often included placing world leaders into videos as dictators, movies such as Downfall (Der Untergang) or Charlie Chaplin’s the Dictator. *“Anyone made a deepfake yet of Putin as Hitler in that Bunker scene from downfall*?*”* However, a significant portion of the requests for deepfakes involved the president of Russia (n = 190). People specifically requested deepfaked pornography, which was seen to humiliate or demean Putin, occasionally involving bestiality (n = 3) and BDSM. *“Would it be possible for some dedicated VFX guy to make a video of Putin*, *heh “developing a connection with a barnyard animal”*?*”* A significant portion also desired to place him into pornography with other men (n = 30). While some justified this as a consequence of Putin’s own anti-LGBT policies, many created an uncomfortable association between sexuality and shame. *“We need to stop this invasion*. *What if we show Russian politicians a high quality deepfake of Putin in an orgy with a ton of men*. *We ask them to stop the war or we will plaster the video all over the internet*. *No offense to gay people*, *but this could stop a war”*. These requests were often accompanied by requests to disseminate and distribute the defamatory video (n = 92), such as hacking Russian news stations or televisions (n = 28).

Some users however did request more positive deepfakes of world leaders which would tell the truth (n = 23), for example to have Vladimir Putin admit to the war crimes carried out by Russian soldiers. *“It’s time for hacker groups to join forces with VFX tech*. *Russia isn’t telling it’s people what’s really happening*, *so we’ll make a deepfake Putin tell them the truth*. *No lies*, *just showing them that civilians are dying and it is a real war”*. While distinctly more noble this was still often accompanied by asking if anyone could hack Russian media to present the deepfake. *“It’s time to play dirty as well*, *we should do a deepfake of the President of Russia*. *We can hack it into Russian news sites and show the real truth of what’s happened during this war”*. This shows a worrying tolerance towards the production and distribution of deepfake disinformation when it fits one’s own political beliefs.

### Deepfake fuelled scepticism

Many tweets in the dataset expressed a healthy scepticism towards deepfakes. People often warned about the dangers of the technology (n = 61) and mentioned how they were preparing for deepfakes (n = 3), and how to identify the technology (n = 7). *“Prepare for Russian deepfake disinformation*. *They are going to use deepfakes to spread propaganda and make excuses for their war crimes”*. Many users engaged in fact checking the media they were consuming and identifying signs that videos were (n = 11) or were not (n = 25) deepfakes. Good media practice was encouraged, tweets promoted healthy scepticism of online information (n = 39), these tweets occasionally highlighted the important work done by government and news agencies in detecting and rebutting fakes. *“Sources say*, *the Russians are planning a deepfake of Zelensky soon where the president will falsely surrender*. *Be prepared”*.

Unfortunately, the majority of this type of Deepfake discourse during the war consisted of unhealthy scepticism fuelled by deepfakes. Fears of deepfakes often undermined users trust in the footage they were receiving from the conflict (n = 85) to the point where they lost trust in any footage coming from the conflict. *“We can’t always trust our eyes anymore*. *Not that this video is fake*, *but all possibilities need to be considered”*. Many Twitter users expressed the opinion that no information from the conflict could be inherently trusted, with the implication that sinister agencies were presenting false narratives. *“None of the videos coming out of this war can be trusted*. *In the next few years*, *the media will be using sophisticated deepfake software*. *If you think we have a problem now*, *wait until we start seeing completely faked videos*, *as opposed to their current lies”*. This quote also highlights the scepticism of journalism and new media (n = 30) as an interpretive repertoire in the dataset. *“This is a western media deepfake*. *These journalists are under the globalists thumb*. *We know what will happen next*. *Praise the lord”*. At its most extreme the anti-media sentiment seemed to be used to justify conspiratorial thinking and a distrust in reliable sources of news. This highlights how deepfake discourse can be used in arguments which undermine the veracity and trustworthiness of news media.

Most emblematic of the epistemic impact of deepfakes were the deepfake accusations, particularly in situations where real media was accused of being deepfake. Tweets in the dataset tended to label real media as fake more (n = 60) often than they correctly fact-checked deepfakes (n = 11). Often this media was of real footage or of footage taken out of context. These accusations were often directed towards an incident where a video of Vladimir Putin appeared to show the Russian President’s hand pass through a microphone. While this was later shown to be a video artifact, a significant number of tweets accused it of being a deepfake. *“Look at his hand passing through the mic*. *This is a possible deepfake and a poorly made one at that*. *The Russian president wasn’t in attendance”*. These claims were often used to justify the theory that Putin was hiding or that he was suffering from serious health issues. *“Vlad’s clearly at deaths door*. *He has had an abdomen surgery and those addresses have been deepfaked”*. Often users would call CGI footage deepfake (n = 12) or confuse the terminology of CGI and Deepfakes (n = 3) when accusing other users of spreading fake news. The word deepfake, took on the role of an adjective in these cases often referring to concepts or people instead of media (n = 15).”*He’s got the world consuming his Deep Fake News spreading his Deepfake ideas and his Deepfake beliefs”*. When used as a buzzword and applied to people and groups, the word deepfake was generally used insultingly. *“This is all nonsense from the Deepfake liberals*, *they’ve made Zelensky into an idol”*. The proclivity of users to call people and governments deepfake and the tendencies to use the word deepfake as a buzzword are a worrying indicator of the lack of awareness of what deepfakes are.

In the most extreme cases of scepticism in the dataset, tweets contained conspiracy theories claiming real world events and individuals were deepfakes. The falsely accused video of Vladimir Putin was one of many examples of deepfake conspiracies theories which focused on deepfaked world leaders (n = 155) or deepfakes being used to represent world leader who were in hiding (n = 30). More broadly, users suggested the war was not as it seems, referring to the entire conflict as a deepfake (n = 21). *“We are being deceived by Ukraine*, *they are probably laughing with Putin over our dollars*. *The war is a deepfake”*. These conspiracies often justified this accusation by relating it to larger criticisms of governments or passing the entire war off as anti-Russia propaganda. *“We need to figure out what is really true*. *Most importantly*, *the truth that Ukraine isn’t real*, *we are being fed lies against Putin*. *All our incompetent leaders in the west*, *under certain payrolls*, *are hiding this from us*. *Look at the Wikipedia page for deepfakes“*. This quote shows how conspiratorial thinking was often related back to broader interpretative repertoires of conspiracies, often referencing interpretative repertoires of conspiracy theories such as deep states and antisemitic conspiracies (n = 10). *“THE SEMITIC DEEPSTATE HAS HAD FREE RUN TO SPREAD THEIR PROPAGANDA AGAINST PUTIN AND THEY MUST BE HELD ACCOUNTABLE”*.

Not all conspiracies were endorsed so wholeheartedly, some users explained the conspiracies as more a humorous thought experiment and reflecting on the implausibility of it. *“The nutty conspiracy theorist in me thinks maybe it isn’t real*. *Either he’s worn the same outfit every day since the first video or he’s been deepfaked”*. Some people created sarcastic conspiracies as a way of criticising other users’ opinions and claims (n = 12).”*Putin was obviously created by a lab in China and will be broadcast to us all using 5G and our vaccines”*. While a lot of these conspiracies were presented earnestly, the outlandishness of these theories often made it hard to judge. Some of the more preposterous conspiracies came from users and in response to media that was seriously posting the conspiracies. The theory that Vladimir Putin had a health issue and was temporarily replaced with a deepfake was picked up as a rumour and ran by a few tabloid newspapers. Some tweets in the dataset then presented this tabloid reporting on online rumours as evidence for the conspiracy. *“This news report suggests that Putin has been using deepfakes for his public appearances*, *which have been pre-recorded to hide his deteriorating health”*.

A less extreme aspect of conspiracy theories in the dataset also involved attributing the creation of the well-known deepfakes to government bodies, for example claiming the two deepfake presidents were made by the opposite governments (n = 33). The two presidential deepfakes were often framed as responses to each other in state sponsored cyber-warfare *“Very cool*. *After Putin’s laughable deepfake of Zelensky*, *the Ukrainians have got their payback with this epic deepfake of Putin*. *Very funny*!*”*. Some conspiratorial accusations in this vein involved claiming that Ukraine and Russia were making the deepfakes of their own presidents so they could claim victimhood (n = 13) *“Does anyone else think that the Russians created the Putin deepfake so it matches the Zelensky deepfake*?*”*.

### Non-misinformation related deepfake discourses

A significant characteristic of non-misinformation related deepfake discourses was humour, both through jokes (n = 84) and through reactions to humorous deepfakes (n = 45). Jokes were used to criticise politicians and to mock conspiratorial deepfake beliefs. Some users made requests for non-serious deepfakes based off current news stories. *“If we made a deepfake of Putin making fun of Jada*, *Will Smith would slap him so hard it could end the war*. *MI6 should get onto this”*. Similarly, humorous deepfakes of the figureheads of the Ukraine war were often used to criticise politicians by placing them into unflattering movie scenes. Users reacted positively to these deepfakes, especially the deepfake of Vladimir Putin being swapped into a violent death scene of the movie Inglorious Bastards. *“There is a great deepfake of Putin getting his head caved in with a bat on YouTube”*. A small number of people found these deepfakes bizarre and in poor taste. Humorous deepfake content when related to the war was framed as embarrassing. *“Someone please get me off this wild ride*, *there’s a deepfake of Putin getting beaten to death in Inglorious bastards while Biden watches”*. In contrast to the requests for demeaning and pornographic content, these requests were not seen as a weapon in the Ukraine war nor did users request them to be used in hacks. Instead, they were viewed as strange indicators of the current state of internet discourse. *“Social media really has the world’s worst opinions on Ukraine*. *There’s videos of people dying soundtracked by folky songs about ww3*, *people stanning Zelensky like Beyonce*, *Deepfake Putin doing TikTok dances”*. Social media companies were also criticised for leaving up violent Putin deepfakes online with some Twitter users believing that they would’ve been taken down if they weren’t of Putin. *“It’s a well-made-deepfake*. *Biden watching Zelensky beating Putin to death with a baseball bat*. *From Inglorious Basterds*. *If it was a video of Biden or Zelensky getting their heads bashed in people would be reporting it to YouTube for removal”*.

References to educational deepfakes also existed in this dataset, consisting of users spreading an educational deepfake with a summary (n = 27). Many of these were positive, especially toward a deepfake of Vladimir Putin soliciting donations for Ukraine. However, reactions to the CGI videos posted by the Ukraine government often expressed distrust towards other media produced by Ukraine (n = 15). “*Look how well made this Ukrainian deepfake is*. *You’d start to lose faith in how real their supposed evidence of Russian involvement in the MH17 plane crash*”. Despite the positive messages of these deepfakes it was felt that by showing people they had the capacities to create fake footage, the Ukraine government undermined their own credibility.

## Discussion

Since computer-generated deepfakes emerged in 2017, researchers have speculated that deepfakes would be used in warfare for propaganda and misinformation [62]. Here we report the first study of deepfakes used in an active war. In line with researchers’ predictions about the harms of deepfakes in war time, there were a great many deepfakes shared online during the period studied. The most notable were the false surrender videos of the Russian and Ukrainian presidents. Though of very poor quality, had these deepfakes been believed, they may have had wide-reaching consequences for the war. In particular, the deepfake of the Ukrainian president undermines the idea that relying on reputable sources will prevent the harms of deepfakes [63], as the video was spread through the hack of a reputable Ukrainian news website. Though there were examples of harmful deepfakes in the war, the majority of deepfakes were not serious attempts to stop or start a war; predictions have severely underestimated the satirical and humorous use of deepfakes. Another important event during the war, the “deepfake” Klitschko video call, indicates the tendency of news articles and governments to label unknown video manipulations as deepfake. In light of the epistemic harms of deepfake discourse, the importance of technical literacy in news reporting around deepfakes is important.

This research had two aims: to study people’s responses online to deepfake content during the Russo-Ukrainian war and to explore if there was practical evidence of the effect of deepfakes on epistemic trust. Our thematic analysis highlighted three main areas of deepfake discourse online; deepfakes and misinformation, deepfake fuelled scepticism and non-misinformation related deepfake discourses. We found evidence for the epistemic harms of deepfakes in cases where people doubted the veracity of real videos and in cases of deepfake conspiracy theories. In the following section we will discuss what these results teach us about how deepfakes are perceived online, and the implications for journalistic practice and future academic research.

### Reactions to deepfakes in the Russo-Ukrainian war

We found that perceptions of deepfakes and deepfake news in the dataset were generally negative. Tweets generally focused on the harms of the technology, expressing fear and shock at the potential harms of the technology similar to the fears expressed in news articles and academic research on deepfakes [64, 65]. Positive discourse around the technology often celebrated the use or potential use of deepfakes in cases where the target was seen as justified. Users often asked for deepfakes to be made of world leaders and to be disseminated through hacks. This evokes past research into deepfake communities on Reddit, where redditors often requested deepfakes for specific purposes [66]. This is indicative of the crowd-sourced nature of deepfakes and how the social demands for certain deepfakes influences what is created and spread [66]. Another element of deepfakes which became apparent through our analysis was the absolute prevalence of deepfake discourse relating to pornography. Past research has estimated that 95% of deepfakes online are pornographic in nature [67]. Despite the Ukraine war showing the potentials of the technology in war and misinformation, there is clearly a widespread tendency to use deepfake technology to create non-consensual pornography, even when relating to the leaders of military conflicts. This harm does not depend on the capacity of deepfakes to deceive it instead was seen as a way to publicly attack a politician’s image. Our research showed people’s desire to shame and humiliate world leaders, but this technology is and will be used, to bully and harass people in their everyday lives [68]. The impacts of harmful defamatory deepfakes and deepfake pornography remain an important and understudied harm of the technology. The prevalence of this harm highlights the need for researchers and policy makers to tackle defamatory deepfakes through research and legislation.

A significant element of deepfake discourse highlighted in our analysis was the humorous and educational discourses around deepfakes. While these have generally been viewed as positive potentials of deepfakes [62], our dataset showed more complex views on the non-misinformation deepfakes. Because they used images of politicians and often related to current events, the humorous deepfakes in the war still served as a way for users to make political commentary through satire and parody. Humorous deepfakes were generally celebrated by users, but many found the videos uncomfortable, especially in instances where the deepfakes were graphic or violent. This highlights the need for more nuanced research into creative uses of deepfakes.

### Deepfakes and distrust

This study provides tentative indications as to how the epistemic damage of deepfakes exist in an online environment and the complex interactions it encourages between truth and falsity existing on social media. The liar’s dividend is the result of an information environment where real information can be easily discredited as fake [19]. Our research provides insight into the creation of an information environment which may potentially lead to the liar’s dividend. Real video and images were decried as deepfake, people often mistook CGI for deepfakes and used deepfakes as a catch-all insult for information they did not like. This shows the practical implications of the previously-studied relationship between awareness of deepfake technology and how it undermines one’s belief in real media [21]. There were also two incidences in our dataset where tweets misidentified real war footage as deepfakes but because of our methodological restrictions on sensitive combat footage we cannot make any claims as to the prevalence of this. While the generally assumed threat of deepfakes is their believability, the labelling of real media as deepfakes shows the capability of deepfake discourse to undermine truth [22]. Though this paper cannot account for pre-existing conspiratorial beliefs or the impact of malicious “bad actors” using deepfakes to encourage scepticism for their own advantage, our results still highlight that deepfakes are being discussed in epistemically harmful ways. We found deepfakes were used for accusations and to cultivate unhealthy scepticism of real media, though future quantitative research is required to highlight both the prevalence of deepfake accusations and its potential effects on trust.

The use of deepfake and CGI educational media by the Ukrainian government was often viewed quite negatively in the dataset. Many users felt that by showing their ability to create deepfakes, the government had undermined the credibility of other authentic video evidence. This has broad implications for educational deepfakes and their potential harms. The current study suggests that educational deepfakes may damage the credibility of the reputable agencies which produce them. By making educational deepfakes, one is only showing the public that their company or agency can produce highly convincing fake videos. Previous quantitative research has shown that educational disclaimers can to some extent mitigate the harms of pure deepfake misinformation [18], but our research indicates that educational deepfakes may still undermine truth in comparison to unedited videos. The effects of educational deepfakes on deepfake related scepticism and the liar’s dividend require further study, but we suggest that governments and organisations that rely on the public’s trust should avoid using deepfakes as part of their messaging.

A novel finding of the analysis was the observed interactions between deepfakes and conspiracy theories. The use of image editing and video editing techniques in conspiracies is not new. The moon-landing conspiracy was fuelled by the current state of Hollywood cinema and visual effects at the time, specifically those used in Stanley Kubrick’s 2001: A Space Odyssey [69, 70]. Deepfakes and the liar’s dividend may provide another tool of deniability to conspiracy theorists. Conspiracy theories harness suspicion of video evidence, which deepfakes have the potential to fuel [71]. Advancement in modern video-editing technologies is likely to inform a host of theories revolving around deepfake replacements of world leaders or world leaders using deepfakes to improve their speeches or to hide their own illnesses. For example, on the 27th of July 2022, a 40 second video of US president Joe Biden in which he did not blink led to many Twitter users to decry the video as a deepfake and endorse similar conspiracies to those that emerged during our analysis [72]. This paper is the first to find evidence of online conspiracy theories which incorporate deepfakes. Conspiratorial deepfake beliefs are a potential problem of deepfakes going forward and one that necessitates further research.

### Limitations

An important limitation of this study is that Twitter may not be representative of other social media platforms. The format of tweets, limited to 280 characters and the prevalence of news sites and bots means the data gathered from the service tends to be less textually dense than information from Reddit or Facebook. Most tweets consisted of short sentences, and longer posts tended to be simple summaries of news articles. The lack of lexical density significantly limited the ability to derive some of the intended meanings, at times it was impossible to gauge if users were being serious or not with their posts. This is an issue that researchers have encountered when studying language, particularly the study of sarcastic language, on Twitter [73]. This was especially true regarding conspiratorial posts where often the ridiculousness of the claim was not indicative of its seriousness. Future research could explore more textually rich online spaces such as online forums and blog posts.

Because the extracted data was from a Western media service, the search terms were in English, and the tweets were in Western European languages, the dataset has the potential for political bias. Telegram and Russian social media were significantly involved in the spreading of anti-Zelensky media and deepfakes which may not be fully represented in our dataset [74]. As such it is impossible for us to make conclusions as to whether the deepfakes and discourse of the crisis were more anti-Russia (as generally observed in our data set) or anti-Ukrainian. Another bias is that the dataset did not include any tweets that had been deleted before data collection so it may not be representative of the misinformation spreading at the time.

A minor limitation of the research is the inability to determine if any of the content was produced by politically motivated “bad actors”. Considering the unverified and anonymous nature of many Twitter users, much of the discourse undermining truth could potentially have been motivated in this manner. However, politically motivated or not, this discourse is still indicative of the real-world social media discourses around deepfake.

One further limitation of this research is the use of the word deepfake in the search terms. This excludes from our dataset incidences where people discussed deepfakes without knowing that they were watching edited footage. Though it would have proved unfeasible to carry out a search for media that people didn’t recognise as being deepfaked, it is still important to note that people who accepted or believed the deepfake media to be real are missing from our dataset, especially in the discussion of people’s ability to be misled by deepfakes.

## Implications

This research has implications into how news media and governments interact with the general discourse around deepfakes. In our dataset, the number of news articles and tweets spreading stories about deepfakes significantly eclipsed actual user discourse. Given that the liar’s dividend is likely to grow with education and efforts to curb deepfakes [2], the dominance of news media in our dataset may give credence to claims that deepfake coverage has outpaced the use of actual deepfakes in misinformation [65]. We need to consider if the news focus on deepfakes is disproportionate to the threat we are currently facing and whether this response is creating more distrust and contributing to an epistemic crisis. Warnings about deepfakes need to be measured against their probability, and due diligence may be necessary before claiming videos which are traditionally faked are actually deepfakes. In many cases, preconceptions and fears about deepfakes informed the anti-media sentiment throughout the dataset. The implication of this is that news media needs to weigh the benefits of pre-bunking and information inoculations against the risks of the liar’s dividend and the undermining of real information. News coverage should also be careful in the labelling of suspected deepfakes, for example news coverage of the dubious supposed deepfake video call with Mayor Klitschko, which possibly only used traditional video fakery, encouraged users to doubt other interactions between politicians and accuse them of being deepfakes. In an information environment where people are endorsing deepfake conspiracy theories and decrying real media as deepfake, it becomes vitally important that news discourse around deepfake encourages literacy in identifying deepfakes and other forms of fakery. News coverage of deepfakes needs to focus on educating people on what deepfakes are, what their potential is, and both what their current capabilities are and how they will evolve in the coming years.

This research has implications as to how deepfakes can be used in warfare. The harms deepfakes have on people’s trust and their concept of truth significantly undermine faith in governments. In our dataset, some users responded negatively to the educational deepfakes, and CGI made by the Ukraine government. While well intentioned, the use of educational synthetic media encouraged Twitter users to frame the real combat footage in Ukraine as deepfakes. The timeline we’ve established for the Ukraine war shows that deepfakes are by the time of writing having real world impacts on the war. The Zelensky deepfake serves as a worrying indicator of the potential harms of the technology. The general playbook against misinformation and deepfakes often encourages people to check the source of any claims or videos. However, the deepfake of Zelensky was distributed in tandem with a hack of both the TV station and news site of a reputable media source. Deepfake literacy and a healthy scepticism of outlandish claims should be encouraged as a way to avoid the harms of deepfakes used in hacked media. This is consistent with research on the protective nature of deepfake literacy and awareness against the harms of deepfake misinformation [75].

In the dataset, almost no tweets involved individuals’ belief in real deepfakes, instead most of the misinformation came from labelling real media as deepfake. The word deepfake tended to devolve into meaning “extra fake” when used as an adjective against individuals, CGI and other non-deepfake media. Deepfakes, already suffering from the lack of consistent definitions, seem to have become a buzzword and an insult. A lot of the deepfake scepticism in our dataset seemed to only have a tentative understanding of what deepfakes were. Non-media objects such as individual people or even the entire war were accused of being deepfakes. The lack of general knowledge of what deepfake means and the tendency to use it towards other fakes has implications for academic researchers. In research that measures proclivity to share deepfakes, the measurements of deepfake sharing are biased by individuals’ perceptions of what deepfakes are. It is possible that if someone has only been presented with a rough definition of deepfake that they may be using the term to describe CGI or traditional fakery. To avoid this, academic research on deepfake sharing behaviours should encourage people describe any deepfakes they have fallen for qualitatively to ensure that they are indeed deepfakes.

## Conclusions

The use of deepfake technologies in the Russo-Ukrainian war is a significant moment in the history of deepfake technologies. For the first time we’ve seen deepfake propaganda and misinformation that has attempted to influence a war. Despite the use of deepfakes in fake surrender videos, the chief focus of deepfake discourse is still the personal defamatory harms of deepfakes. Individuals tended to overlook or even encourage the harms of defamatory deepfakes when they were directed towards political rivals. The current research provides the first practical qualitative evidence of the of the epistemic harms of deepfakes on social media. In the dataset, real videos were accused of being deepfakes and deepfakes fuelled conspiratorial beliefs and unhealthy scepticism. The use of deepfakes in education and entertainment must also be reconsidered in light of the epistemic harms of deepfake technology.

## Supporting information

S1 Data (CSV)
